# Lactate Metabolism-Associated lncRNA Pairs: A Prognostic Signature to Reveal the Immunological Landscape and Mediate Therapeutic Response in Patients With Colon Adenocarcinoma

**DOI:** 10.3389/fimmu.2022.881359

**Published:** 2022-07-11

**Authors:** Junbo Xiao, Xiaotong Wang, Yajun Liu, Xiaowei Liu, Jun Yi, Jiuye Hu

**Affiliations:** ^1^ Department of Gastroenterology, Xiangya Hospital, Central South University, Changsha, China; ^2^ Hunan International Scientific and Technological Cooperation Base of Artificial Intelligence Computer Aided Diagnosis and Treatment for Digestive Disease, Xiangya Hospital, Central South University, Changsha, China; ^3^ National Clinical Research Center for Geriatric Disorders, Xiangya Hospital, Central South University, Changsha, Hunan, China; ^4^ Department of Gastroenterology, Affiliated Hospital of Xiangnan University, Chenzhou, China

**Keywords:** lactate metabolism-related lncRNAs, prognosis, tumor immune cell infiltration, therapy response, colon adenocarcinoma

## Abstract

**Background:**

Lactate metabolism is critically involved in the tumor microenvironment (TME), as well as cancer progression. It is important to note, however, that lactate metabolism-related long non-coding RNAs (laRlncRNAs) remain incredibly understudied in colon adenocarcinoma (COAD).

**Methods:**

A gene expression profile was obtained from the Cancer Genome Atlas (TCGA) database to identify laRlncRNA expression in COAD patients. A risk signature with prognostic value was identified from TCGA and Gene Expression Omnibus (GEO) cohort based on laRlncRNA pairs by the least absolute shrinkage and selection operator (LASSO) and Cox regression analyses. Quantitative real-time polymerase chain reaction (qRT-PCR) and functional experiments were carried out to verify the expression of laRlncRNAs in COAD. The relationship of laRlncRNA pairs with immune landscape as well as the sensitivity of different therapies was explored.

**Results:**

In total, 2378 laRlncRNAs were identified, 1,120 pairs of which were studied to determine their prognostic validity, followed by a risk signature established based on the screened 5 laRlncRNA pairs. The laRlncRNA pairs-based signature provided a better overall survival (OS) prediction than other published signatures and functioned as a prognostic marker for COAD patients. According to the calculated optimal cut-off point, patients were divided into high- and low-risk groups. The OS of COAD patients in the high-risk group were significantly shorter than that of those in the low-risk group (P=4.252e-14 in the TCGA cohort and P=2.865-02 in the GEO cohort). Furthermore, it remained an effective predictor of survival in strata of gender, age, TNM stage, and its significance persisted after univariate and multivariate Cox regressions. Additionally, the risk signature was significantly correlated with immune cells infiltration, tumor mutation burden (TMB), microsatellite instability (MSI) as well as immunotherapeutic efficacy and chemotherapy sensitivity. Finally, one of the laRlncRNA, LINC01315, promotes proliferation and migration capacities of colon cancer cells.

**Conclusion:**

The newly identified laRlncRNAs pairs-based signature exhibits potential effects in predicting prognosis, deciphering patients’ immune landscape, and mediating sensitivity to immunotherapy and chemotherapy. Findings in our study may provide evidence for the role of laRlncRNAs pairs as novel prognostic biomarkers and potentially individualized therapy targets for COAD patients.

## Introduction

Colorectal cancer (CRC), a cancer of the gastrointestinal tract, is one of the top five cancer diagnosed in China (1) and ranks third among cancer-related deaths worldwide (2, 3). The category includes colon cancer and rectal cancer, both of which are highly aggressive and prevalent cancer types. Global rates of colon adenocarcinoma (COAD) are rising at a rate of 4% or more each year, which underscores the burden this disease on health throughout the world (4). The availability of advanced treatment options, such as chemotherapy, immunotherapy and colonoscopy screening, has contributed to the longevity of COAD patients; and 64% of them survive beyond their first five years after diagnosis (5). As surgical intervention is available for early COAD, a large majority of patients with advanced COAD suffer from a poor therapeutic outcome with higher rates of malignant recurrence and distant metastases, resulting in a 5-year survival rate of less than 10% (6, 7). Immune checkpoint genes (ICGs), such as PD-1/L1, have been identified to be highly efficacious in the treatment of multiple types of cancer (8, 9). However, only a limited number of COAD patients can benefit from the current checkpoint immunotherapy. As a result, identifying tumor prognostic indicators, as well as understanding the molecular mechanisms that lead to COAD, are imperative for assessing tumor progression and forecasting the effects of therapeutics effectiveness following disease onset.

In the wake of Warburg effect (10), lactate was once perceived as an innocuous metabolic by-product (11). New lines of studies have revealed, however, that excessive lactate accumulation by cancer cells, a critical hallmark of cancer (12–15), can lead to an acidified tumor microenvironment (TME) (16). It may be favorable for tumor development and metastasis and hinder function of immune cells such as dendritic cells (DCs) and macrophages (17, 18). Emerging studies indicate that lactate metabolism may have pleiotropic effects on tumorigenesis, affecting a range of factors such as TME, patients’ survivability, and immune surveillance escape (19, 20). It is noteworthy that an acidic TME could impede some therapeutic drugs’ extracellular accumulation, which normally penetrate into cells *via* passive diffusion (21, 22). To be fair, these findings suggest that targeting lactate metabolism in tumors might be a promising choice for cancer treatment. At this stage, further work is still needed to understand the role of lactate metabolism in COAD and its impact on immune regulation, as well as its mechanism of exerting a synergistic role with current immunotherapies.

Long non-coding RNAs (lncRNAs) are transcripts with a sequence of nucleotides longer than 200, which are incapable of encoding proteins (23, 24). Instead, lncRNAs are involved in a variety of biological processes, including cell proliferation, migration, apoptosis, and metastasis (25–27). A growing body of evidence suggests that lncRNAs are critical to the development and progression of COAD, whose dysregulations, however, may potentially affect cancer prognosis and clinical therapeutics outcome (28, 29). Xu et al. proposed that lncRNA SATB2-AS1 modulates the density of immune cell and TH1 chemokine expression in the TME, ultimately affecting tumor metastasis and patients’ prognosis (30), offering insights into novel biomarkers and treatment targets related to lncRNAs in CRC. Additionally, prior studies have demonstrated a significant predictive and prognostic value for lncRNA-associated signature among cancers. Based on a set of eight immune-related lncRNAs, Zhu et al. evaluated the ICGs’ efficacy in treating patients with esophageal squamous cell carcinoma (31). According to Wei et al.’s study, their signature for CRC included eight autophagy-related lncRNAs and predicted adverse outcomes with a value of 0.689 for the area under the curve (AUC) (32). Nonetheless, this method was not an entirely perfect tool for prefiguring prognoses. The lncRNA signatures have a serious limitation since specific expression levels must be specified and even normalized to prevent batch effects across different platforms. It has also been shown that the combination of two biomarkers results in the establishment of a more accurate predictive model (33, 34). This novel algorithm allowed Hong et al. to construct a prognostic model that incorporated immune-related lncRNAs for hepatocellular carcinoma patients with AUC values of 0.865, 0.851, and 0.904 after 1 year, 3 years, and 5 years, respectively (35).

However, there is still relatively few research describing lactate metabolism-related lncRNA (laRlncRNA) pair signature applicable for prognosis prediction in COAD patients. This study was hence conducted to investigate the utility of laRlncRNA pairs signature, independent of specific expression levels, in screening survival outcome, elucidating immunological landscape, as well as forecasting their interactions with immunotherapy and chemotherapy in COAD patients.

## Materials and Methods

### Acquisition of Data

**Figure 1:**
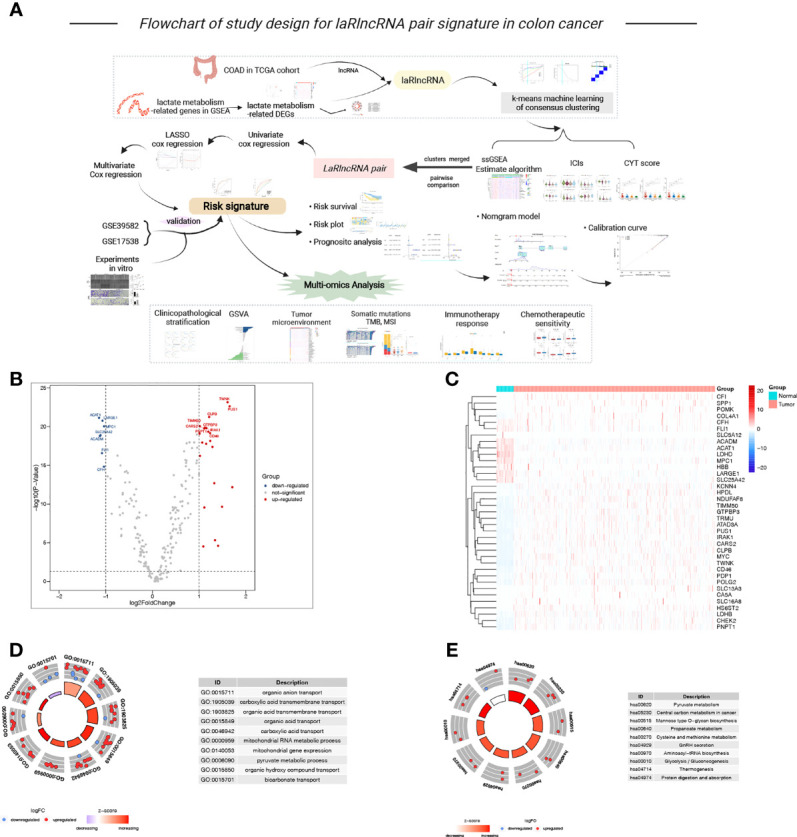
**(A)** Flowchart of our study, **(B, C)** The volcano plot and heatmap of lactate metabolism-related DEGs between tumor and normal samples, **(D, E)** The GO and KEGG circle plot of functional enrichment analysis.

Data from RNA-sequencing (RNA-seq) was accessed, along with clinical details pertinent to the study available through The Cancer Genome Atlas (TCGA) database (https://portal.gdc.cancer.gov/), containing 480 tumor and 41 normal samples. A notable fact was that only those patients who were followed up for a period longer than one month were used for the study. Detailed information about the clinical characteristics of patients with COAD are summarized in **Supplementary Material S1**. There were 823 COAD patients in the GSE39582 and GSE17538 datasets, which were extracted from the GEO database (http://www.ncbi.nlm.nih.gov/geo/). To remove batch effects of the two GEO cohorts, sva package and the ComBat algorithm were applied for further analysis (36). A TCGA dataset was employed to form the training cohort, whereas the merged GEO dataset served as the validation cohort. **Figure 1A** depicts an overview of our study design *via* BioRrender (https://app.biorender.com/).

### Identification of Lactate Metabolism-Related Genes and laRlncRNAs

A total of 227 lactate hallmark genes were downloaded from the Gene Set Enrichment Analysis (**GSEA)** (http://www.gsea-msigdb.org/gsea/index.jsp) (37)** (** **Supplementary Material S2** **).** In subsequent analyses using the “limma” R package, lactate metabolism-related differentially expressed genes (DEGs) were screened out between the normal samples and the tumor samples and plotted *via* volcanoes and heatmaps, with logfold change (|logFC|) > 1 and false discovery rate (FDR) < 0.05 used as thresholds for comparing expression differences. Then, functional enrichment analysis [Gene Ontology (GO) and Kyoto Encyclopedia of Genes and Genomes (KEGG)] were carried out to explore the potential biological attributes and molecular function of these DEGs (38). Correlation analyses were implemented through loop iteration to calculate the correlation coefficients and p values between each lncRNA in TCGA cohort with individual lactate metabolism-related DEG with the threshold values of |Pearson R| >0.3 and P < 0.001 to identify candidate laRlncRNAs (39).

### Unsupervised Consensus Clustering of COAD Molecular Subtypes Using laRlncRNAs

With the “ConsensusClusterPlus” R package from Bioconductor, an unsupervised consensus clustering was performed based on the list of laRlncRNAs that had been obtained in the preceding step, using the k-means machine learning algorithm. In clustering, variable “k” represents the number of clusters. It was to divide or estimate patterns of lactate regulation among cases into various molecular subtypes for further analysis (40). At the same time, the process was repeated 1,000 times. The optimal number of clusters k was determined by considering where the magnitude of the cophenetic correlation coefficient decreased (41). Then, single-sample gene set enrichment analysis (ssGSEA) was conducted to assess the abundance of different immune features (42). It was calculated according to the proportion of immune cells and stromal cells using ESTIMATE to compute the immune score, stromal score, estimate score, and tumor purity (43). Ten common ICGs were then compared in different clusters, including PDCD1 (PD-1), CTLA4, CD80, CD86, CD274, LGALS9, NECTIN2 (44, 45), PDCD1LG2, PVR (46), and TNFSF14. The CYT score, a measure of cytolytic activity by immune cells, was calculated on the basis of the geometric mean expression level of the genes GZMA and PRF1 (47). With a higher CYT score, the expressions of ICGs, such as CD80, CD86, PD-L1/L2, LGALS9 and TNFSF14 grew gradually, providing another benchmark for the selection of immune checkpoint therapy (48, 49).

### laRlncRNA Pairs Construction

A pairwise comparison of overlapping laRlncRNA expression profiles derived from both TCGA and GEO datasets was conducted. Utilizing the R software *via* FOR loop function, randomly pair and examine the expression of laRlncRNA A and laRlncRNA B in each COAD sample. The algorithm presents a scoring system in which the score of laRlncRNA pair is 1 if the expression level of the laRlncRNA A is higher than that of the laRlncRNA B; otherwise, it is 0, resulting in the construction of a 0-or-1 value of a gene pair matrix in this way (50). The score of a laRlncRNA pair was 0-or-1 in <10% or >90% of the samples in either the training or validation set, the laRlncRNA pair was deemed invalid (34). Following this screening, the remaining pairs were used for subsequent investigations.

### Development of a Prognostic Signature Using laRlncRNA Pairs

To identify laRlncRNA pairs with prognostic significance, a univariate Cox regression analysis was performed for the training set (FDR < 0.05), associated with the Least absolute shrinkage and selection operator (LASSO) analysis to prevent overfitting of the prognostic signature. It was determined that laRlncRNA pairs-based prognostic signature possesses a higher stability through a multivariate Cox regression analysis using the survival, survminer, and glmnet R packages (FDR < 0.05). Calculation of the prognostic signature risk score was carried out by multiplying the expression level by the Cox regression coefficients obtained from laRlncRNA pairs following the formula: $\text{risk score}=\sum_{i=1}^{n}\text{βi}*\text{λi}$ , with n symbolizing the numbers of laRlncRNA pairs linked together with the signature construct, and βi and λi referred to the regression coefficient and the 0-or-1 value of a laRlncRNA pair, respectively. According to the optimal cut-off point of the risk score, patients were categorized into high- and low-risk groups using the surv_cutpoint function of the survminer package. An analysis of comparing the difference of survival rates between the two risk groups was conducted using Kaplan-Meier (KM) and log-rank statistical methods. Both univariate and multivariate Cox regression analyses were then performed to ascertain whether the risk score could be an independent predictive factor for the prognosis of COAD patients. The survival predictors for 1, 3 and 5 years were generated by using a nomogram derived from clinicopathological factors. Through calibration graphs, the differences between the nomogram-predicted and the actual survival rates were evaluated and identified by overlapping with the reference line as proof of the accuracy of the model. To assess the prediction accuracy and compare the constructed signature and clinical characteristics of the training cohort, receiver operating characteristic (ROC) curve and AUC values were served as predictive indicators. Finally, AUCs of this laRlncRNA pairs-based prognostic signature were compared with those of other published signatures from TCGA database.

### Validation of the Signature of laRlncRNA Pairs

The GEO validation cohort was recruited to test the validity of the signature developed from the TCGA training cohort. Stratifying patients from GEO sets into high- and low-risk groups was done based on the cut-off point of the risk score of the training set. Survival analysis and Cox regression analysis were then applied to find whether the laRlncRNA pairs-based signature was significantly associated with overall survival. Moreover, the clinicopathologic features and risk score were utilized to perform both univariate and multivariate Cox analyses in GEO datasets to uncover prognostic factors associated with COAD.

### Colon Cancer Tissues, Cell Culture, RNA Extraction and Quantitative Real-Time Polymerase Chain Reaction

Our team collected 4 cases of colon cancer along with adjacent normal tissue specimens from the Department of General Surgery at Xiangya Hospital, Central South University, to further verify the expression of lncRNAs in our signature.

The human normal intestinal epithelial cell line NCM460 as well as colon cancer cell lines SW480, HT29, HT116, CACO2, HCT15 were donated by the Cancer Research Institute of Central South University (Hunan, 243 China). Cells were cultured in DMEM or RPMI-1640 medium (Gibco Laboratories, Grand Island, NY, USA) in an incubator containing 5% CO2 at 37°C supplemented with 10% FBS,100 U/mL penicillin and100 μg/mL streptomycin.

Total RNA was extracted from the cultured cells using TRIzol reagent (Invitrogen, Carlsbad, CA, USA). Then, 1 μg of total RNA was reversely transcribed using the PrimeScriptTM RT reagent Kit (TransGen Biotech,Beijing,China). Quantitative real-time PCR (qRT-PCR) was performed with the QuantStudio™ 5 System (Thermo Fisher Scientific, USA) using the SYBR-Green PCR Master Mix (TransGen Biotech,Beijing,China). The qRT-PCR conditions comprised initial denaturation for 30 s at 95°C, and 40 cycles of for 5 s at 95˚C, and for 15 s at 60˚C, and for 10s at 72°C. Primers were used and described **in** **Supplementary Material S3**. The relative expression levels of laRlncRNAs were calculated by the 2- ΔΔCt method (**Supplementary Material S4**).

### Transfection

The small interfering RNA (siRNA) targeting LINC01315 for downexpression, and the pcDNA3.1 plasmid vector for overexpression was synthesized from GENERAL BIOL (Anhui, China). Both were transfected by Lipo2000™ (Invitrogen,Carlsbad, CA), into the cell lines following the manufacturer’s protocol. The siRNA was also described **in** **Supplementary Material S4**.

### Cell Proliferation Assays

In the 96-well plates, an overall number of 1*10^3^ transfected cells were seeded for 12 hours, 24 hours, 48 hours, and 72 hours. The cell counting kit 8 (CCK-8, APExBIO, Houston, USA) was chosen, and each well was filled with 10 μL of CCK-8. Lastly, 450 nm wavelength was used to measure the absorbance of each well after incubation for 1 hour at 37°C.

### Migration Assay

The upper chamber was seeded with 2*10^5^ cells in 200 μL serum-free medium, while the lower chamber was plated with 600 μL of 10% fatal bovine serum medium. Cells not penetrating the membrane were removed with cotton swabs, while migrating or encroaching cells were fixed with 0.1% crystal violet after incubation for 24 hours at 37 °C with 5% CO2.

### Wound Healing Assay

In 6-well plates, cells were plated at a seeding destiny of 1×10^6^/well and then cultured to 90% confluence, after which cell layers were scratched with sterile 100 µl pipette tips to creat wounded gaps. The plates were gently dished with PBS and cultured for 48 hours. At the indicated time points, wound gaps were photographed.

### Gene Set Variation Analysis of Different Risk Groups

GSVA with “GSVA” R packages was conducted to figure out the differences in the biological and molecular characteristics that differentiated high-risk and low-risk groups (51). GSVA scores were calculated by using the MSigDB database (v7.1 updated in March 2020; https://www.gsea-msigdb.org/gsea/msigdb/index.jsp), which contains over 20,000 gene sets (52). By using the hallmark gene sets, “c2.cp.kegg.v6.2.symbols.gmt” and “h.all.v7.2.symbols.gmt” as the reference gene set, 25 functional pathways were finalized and visualized to reveal the most significant correlations with different risk groups in COAD.

### Infiltration Analysis of Immune Cells

A method was developed to elucidate the relationship between risk groups according to the laRlncRNA pairs-based signature and the relative abundance of tumor-infiltrating immune cells (TIICs) using datasets including ESTIMATE (43), ssGSEA (42), TIMER (53, 54), QUANTISEQ (55, 56), MCPCOUNTER (57), EPIC (58), CIBERSORT ABS (59), and CIBERSORT (60, 61). The relative content of TIICs under different algorithms was visualized by using the heatmap. Aside from that, correlation analysis of the risk score and the infiltration density of six TIICs, such as B cell (62), macrophage (63), myeloid dendritic cell (64), neutrophil cell (65), T cell CD4+ and T cell CD8+ (66, 67), was performed to demonstrate the immunologic features based on TIMER.

### Analysis of Somatic Mutations, Tumor Mutation Burden, Microsatellite Instability, and ICGs Expression Among Different Risk Groups

The mutation annotation format (MAF), obtained from the TCGA database, was used to compare somatic mutations between different risk groups of COAD patients *via* maftools package (68). Evidence has indicated that patients with a higher TMB are more likely to benefit from immunotherapy owing to the existence of a greater number of neoantigens (69). MSI signifies a situation that new alleles occur in a tumor caused by alterations in a microsatellite length and is reported to be reckoned as a potential hallmark of immune-checkpoint-blockade therapy (70). In this study, we calculated TMB scores as the number of all nonsynonymous mutations/exon length (35 million) for each COAD sample **(** **Supplementary Material S5** **)**. COAD patients were classified into different MSI scores: microsatellite stable (MSS), MSI-low (MSI-L), and MSI-high (MSI-H) by TCGA project **(** **Supplementary Material S6** **)**. Then, both TMB and MSI scores were compared between the two risk groups.

Seven ICGs were chosen to assess the differences of their expression levels in the high- and low-risk groups, including CD274 (PD-L1), cytotoxic T-lymphocyte antigen 4 (CTLA-4), mucin domain-containing molecule 3 (TIM-3; HAVCR2), indoleamine 2,3-dioxygenase 1 (IDO1), lymphocyte-activation gene 3 (LAG3), programmed death 1 (PD-1/PDCD1) and its ligand 2(PD-L2) (71, 72). The relationship between ICGs and risk score were tested using Spearman’s correlation coefficient to investigate the potential immunotherapeutic implications of the laRlncRNA pairs-based signature.

### Investigation of Differences in Chemotherapeutic Efficacy

A drug’s IC50, or half of its maximum inhibitory concentration, indicates the amount of drug required to inhibit 50% of cancer cells. Accordingly, corresponding IC50 values were calculated by the R package “pRRophetic” (73) to evaluate the significance of the laRlncRNA pairs-based signature among six types of chemotherapeutics (i.e., camptothecin, doxorubicin, erlotinib, gemcitabine, paclitaxel, and rapamycin) when applied to the treatment of patients with COAD. IC50 was compared between high- and low-risk groups using the Wilcoxon signed-rank test. As a result, boxplots, utilizing the ggpubr, pRRophetic, and ggplot2 R packages, are displayed, and a cutoff value of P < 0.05 was determined.

### Statistical Analysis

Statistical analysis was performed using the R software (version 3.6.3 https://cran.r-project.org/) and Perl software (version 5.30 https://strawberryperl.com/). Heatmaps of clusters and maps of volcanoes were created by using gplots and heatmap packages. Cox proportional hazards regression analyses were conducted both on a univariate and multivariate basis using the survival package. As a part of the analysis, stratification analysis was used to determine the clinicopathological relevance of the defined risk score regarding age, gender and TNM stage. A P value < 0.05 indicates statistical significance.

## Results

### Lactate Metabolism-Related Genes and laRlncRNAs

Among 227 lactate metabolism-related genes, 37 DEGs in COAD patients of TCGA cohort were identified with FDR< 0.05 and logFC >1, 27 of which were up-regulated (TRMU, TIMM50, POLG2, CHEK2, CFI, CARS2, LDHB, GTPBP3, NDUFAF8, IRAK1, PNPT1, CLPB, CD46, ATAD3A, PDP1, COL4A1, POMK, SLC16A8, HPDL, TWNK, PUS1, KCNN4, MYC, CA5A, HS6ST2, SPP1 and SLC13A3) and 10 were down-regulated (SLC5A12, LDHD, HBB, ACAT1, ACADM, SLC25A42, FLI1, LARGE1, CFH and MPC1). Volcano and heatmap representations of lactate-related DEGs are provided **in** **Figures 1B, C**. To provide insight into the biological functions and pathways involved in these lactate metabolism-related DEGs, the GO enrichment analysis revealed that these DEGs mainly participated in organic acid transport, carboxylic acid transport, pyruvate metabolic process, and anion transmembrane transporter activity, etc. **(** **Figure 1D** **)**. **Figure 1E** illustrates the KEGG pathway enrichment analysis, such as central carbon metabolism in cancer, mannose type O−glycan biosynthesis, pyruvate metabolism, glycolysis/gluconeogenesis and some cancer-related signaling pathways. Afterwards, 14,142 lncRNAs were obtained from the TCGA training cohort, of which 2378 laRlncRNAs were identified through correlation analysis (|Pearson R| >0.3 and P < 0.001; **Supplementary Material S7**).

### Unsupervised Consensus Clustering of laRlncRNAs in the Classification of COAD Subtypes

**Figure 2:**
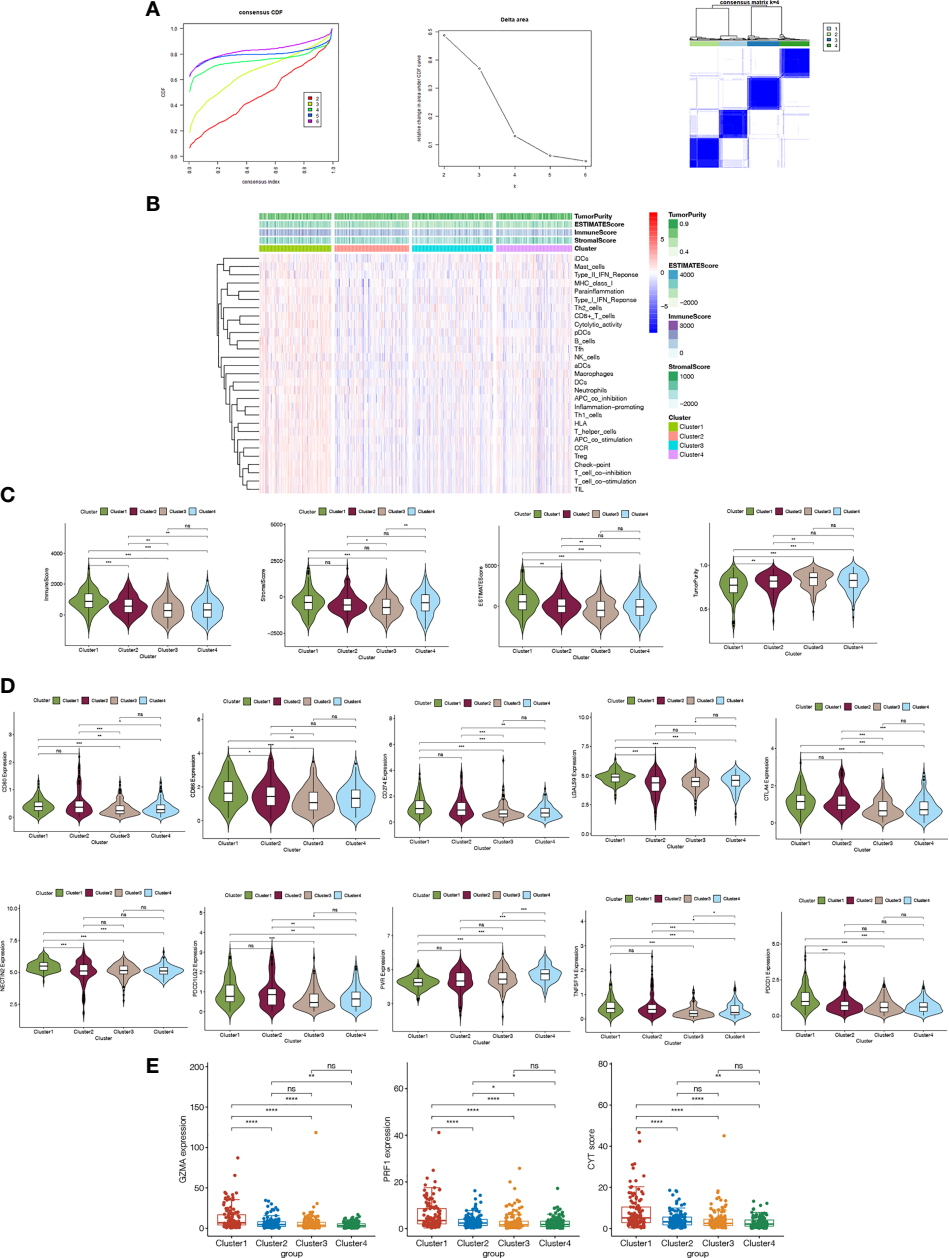
**(A)** Consensus clustering CDF of k=2 to 6; Delta area under the CDF curve; Consensus clustering matrix of k=4. **(B)** Heatmap illustrating the relationships of four clusters and different immnue features **(C)** Immune, stromal, estimate scores and tumor purity using ESTIMATE **(D)** The expression of immune checkpoint genes in different clusters **(E)** The correlations of GZMA, PRFI gene expressions and CYT scores with clusters.

As a first step toward exploring the relationship between expression of laRlncRNAs and COAD subtypes in TCGA, the ConsensusCluserPlus R package was employed to conduct consensus clustering analysis. In accordance with the empirical cumulative distribution function (CDF) plot and delta area plot, k=4 was found to provide optimal cluster stability, illustrating four clustering patterns for COAD patients, namely Cluster1 (n=114), Cluster2 (n=118), Cluster3 (n=127), and Cluster4 (n=120) (**Figure 2A**).

The involvement of ICGs has been reported in colon cancer development (74, 75). We further pondered whether any differences were observed regarding ICGs expression and TIICs to investigate the immunological characteristics of COAD. The CIBERSORT algorithms were used to explore associations between TIICs and the four clusters. **Figure 2B** shows a comparison regarding enrichment levels of 25 immune features across clusters. Using the “ESTIMATE” package, the estimate, immune, and stromal scores were calculated to compare the TME between different clusters. Compared to the other three groups, cluster 1 had significantly higher immune and stromal scores, while cluster 3 and 4 did not show any significant difference in estimate or immune scores. The stromal score and estimate score were the lowest in cluster 3 and the highest in cluster 1 (**Figure 2C**). As the results shown in **Figure 2D**, expression differences of the ten ICGs we selected across the clusters were analyzed and it was found with wide variations. In particular, the expression levels of these genes, including PDCD1, in cluster 1 differed significantly with cluster 3 and 4; clusters 3 and 4 exerted no significant correlations among CD80, CD86, CD274, NECTIN2, and PTCDLG2; the expression levels of ICGs such as CD80 and TNFSF1, were the highest in cluster 2, indicating that cluster 2 might have a better response to ICGs-targeted immunotherapies. Additionally, CYT score, an indicator of immune cytolytic activity, was represented by the genes of GZMA and PRF1. The results in **Figure 2E** showed that cluster 1 had the highest CYT score and was significantly correlated with the other three clusters, respectively. Moreover, we validated the results of **Figure 2** in the GEO cohort (**Figure S1**). Taken together, the consensus clustering analysis of laRlncRNAs was successful in highlighting the different immune characteristics of COAD molecular subtypes. It was found to be significantly related to the intensity of immune infiltration and might be useful in assessing the response to immunotherapy of COAD patients.

### Identification of laRlncRNA Pairs-Based Prognostic Signature

**Figure 3:**
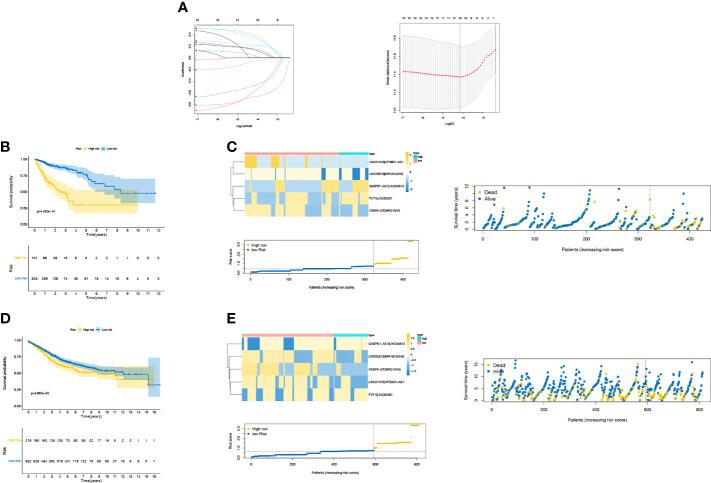
**(A)** LASSO Cox regression analysis. Kaplan—Meier (KM) curve for overall survival (OS) of COAD patients in different risk group, risk survival status plot in TCGA cohort **(B, C)** and GEO cohort **(D, E)**.

Our previous study found that cluster 1 differed significantly from the other three clusters in terms of immune score and stromal score, and the expression levels of ICGs in cluster 1 also showed significant difference with other clusters. We sought to identify laRlncRNAs responsible for these significant differences between different COAD clusters for further study. So, we carried out differential expression analyses on cluster1 - cluster2, cluster1 - cluster3, and cluster1 - cluster4, and then overlapped their differentially expressed laRlncRNAs, from which 562 laRlncRNAs shared in the training cohort and validation cohort **(** **Supplementary Material S8** **)**. A pairwise comparison was performed with the algorithm described in “Methods” to generate a score for each laRlncRNA pair for further analysis. As outlined by univariate Cox regression analysis, 15 out of 1,120 pairs of laRlncRNAs served as potential prognostic indicators (P < 0.05, **Supplementary Material S9**). As shown in **Figure 3A**, the set was subject to LASSO Cox regression analysis to avoid overfitting, and 10 out of 15 laRlncRNA pairs were chosen as the appropriate candidates for constructing a risk signature. Multivariate Cox regression eventually established a laRlncRNA pairs-based signature for patients with COAD based on 5 laRlncRNA pairs (**Supplementary Material S10**). Additionally, laRlncRNAs in our signature were also significantly correlated with the lactate metabolism pathway in GSEA, as shown in **Figure S2**.

**Figure 4:**
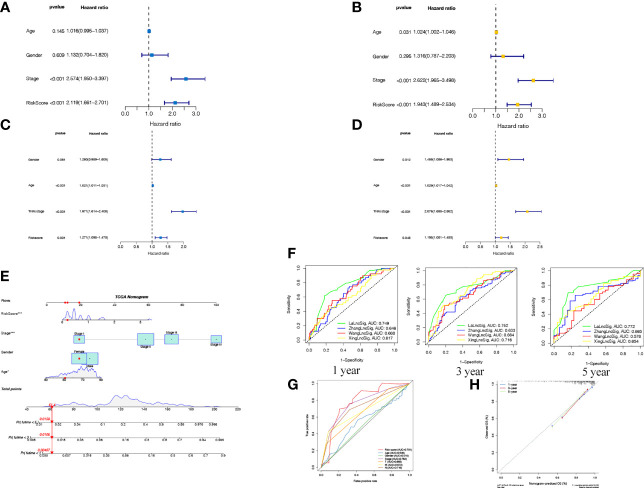
Cox analysis in TCGA **(A** univariate **B** multivariate**)** and GEO **(C** univariate **D** multivariate**)** showed that the signature was an independent risk factor for COAD patients. **(E)** A nomogram established regarding the risk score and clinicopathological charateristics. **(F)** ROC curves of laRlncRNA pairs-based signature at 1, 3, 5-year compared with three other lncRNA-based signature studies on COAD patients. **(G)** The AUC values of the risk score and clinicopathological features. **(H)** Calibration plot to depict the consistence between the predicted and the actual OS at l, 3, 5 years.

The COAD patients were classified into high- and low-risk groups according to the optimal cut-off point of 0.726. The survival curve in **Figure 3B** was employed to examine the differences in survival between the two risk groups. Patient risk scores were significantly related to overall survival of patients in the TCGA set, as those in the high-risk group had a higher risk of mortality (P <0.001). The survival status plot in **Figure 3C** showed that there was an inverse relationship between the risk score of patients and their survival rate. Moreover, the risk heatmap showed positive correlation between 5 laRlncRNA pairs and risk levels. These results were then used to calculate the AUC values to assess whether the signature could successfully predict the overall survival of COAD patients in the training cohort. The AUCs of our laRlncRNA pairs-based signature for 1, 3, and 5 years were 0.749, 0.752and 0.772, respectively, whereas those obtained in three other lncRNA-based signature studies on COAD patients were much lower, which were ZhangLncSig (76), WangLncSig (77) and XingLncSig (78), respectively in **Figure 4F** **(** **Figure S3** **showed the GEO cohort validation results)**. In comparison to other traditional clinical pathological variables, the AUCs of our risk signature showed great accuracy in predicting prognosis of patients with COAD **(** **Figure 4G** **)**. Additionally, univariate (**Figures 4A**) and multivariate (**Figures 4B**) Cox regression analyses were also done to identify factors that significantly affected the prognosis of COAD patients. It was found that the risk signature could serve as an independent predictor of prognosis (p < 0.001, HR =1.943, 95% CI [1.489–2.534]), as well as stage (p < 0.001, HR = 2.622, 95% CI [1.965–3.498]).

An approach by which 1-, 3-, and 5-year survival rates could be more accurately predicted was to construct a nomogram model based on cox regression results (**Figure 4E**), which included age, gender, stage of disease, and risk score. On top of that, the calibration curves in **Figures 4H** comparing the predicted and actual survival rates of COAD patients indicated that the predicted survival rates were in good agreement with those actual rates (C-index = 0.821), confirming the accuracy of this nomogram model.

### Signature Validation of laRlncRNA Pairs

A combat function in the ‘sva’ package properly corrected batch effects from different cohorts of GSE39582 and GSE17583 datasets. The risk score for laRlncRNA pairs were established by using the previous formula as well. A cut-off point of 0.726 for the risk score of the validation cohort was the same as that of the training cohort. Intriguingly, there was also a statistically significant association between this signature and the overall survival of COAD patients (P = 2.865e-02, **Figure 3D**). Based on the survival analysis, patients in high-risk groups had a significantly lower overall survival than those in low-risk groups. The plots of risk scores and survival times also indicated clearly that survival rates and survival times declined with the increase of risk scores (**Figure 3E**). Meanwhile, both univariate (**Figure 4C**) and multivariate (**Figure 4D** **)** Cox regression analyses indicated that laRlncRNA pairs-based signature served as an independent prognostic factor (P < 0.05, HR =1.198, 95% CI [1.001–1.433]), implying that the risk signature developed from training cohort was highly efficacious and robust.

### Subclinical Stratification Analysis

**Figure 5:**
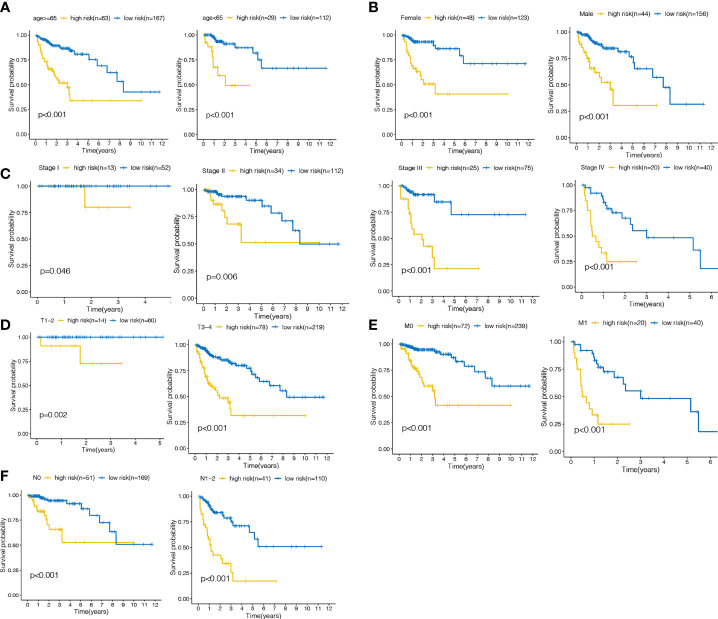
The survival curves of the lalncRNA pairs signature concerning each strata of age, gender, TNM stage. **(A)** ≥65 years, <65years **(B)** Female, male **(C)** stage I, II, III, IV **(D)** T1-2, T3-4 **(E)** M0,M1 **(F)** N0,N1-2.

Clinicopathological stratification analysis of the training cohort was performed, including age, gender, grade and TNM stage. The laRlncRNA pairs-based signature remained significantly correlated with poor survival regardless of older (≥65 years) or younger (<65 years), female or male, stage T 1-2 or T 3–4, M0 or M1 and N0 or N1-2 patients (all P < 0.05; **Figures 5A–F**), indicating that the detection of laRlncRNA pairs-based signature in accordance with risk stratification may serve as a reliable tool for predicting COAD survival based on subclinical stratification by age, gender, and TNM stage. Furthermore, we validated the results of GEO cohort in **Figure S4**.

### Signaling Pathways Mediated by the laRlncRNA Pairs-Based Signature

**Figure 6:**
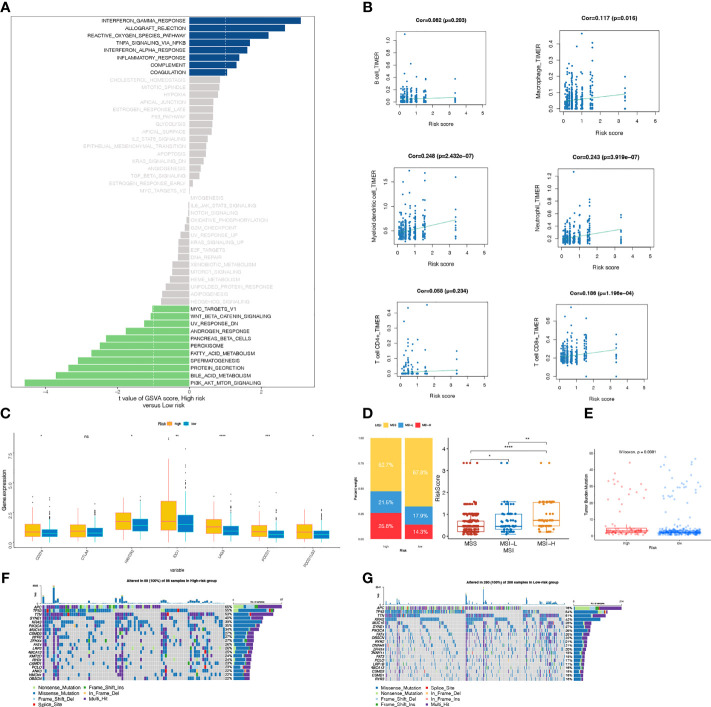
**(A)** GSVA analysis **(B)** Spearman correlation analysis between the signature and six immune cells (B cell, Macrophage, Myeloid dendritic cell, Neutrophil cell, T cell CD4+ T cell CD8+) ; **(C)** The comparison of PDCDl, PDCD1LG2, LAG3, HAVCR2, CD274,IDO1 and CTLA-4 expression levels between high-risk and low-groups; **(D)** Relationships between risk score and MSI. **(E)** Relationships between risk score and TMB. **(F, G)** The waterfall plot of somatic mutation landscape between two risk groups, ranked by top 20 frequently mutated genes.

GSVA analysis was employed to investigate the underlying biological mechanisms of the laRlncRNA pairs-based signature in the progression of COAD. A total of 25 functional pathways were identified as being associated with different COAD risk groups, among which INTERFERON_GAMMA_RESPONSE, ALLOGRAFT_REJECTION, REACTIVE_OXYGEN_SPECIES_PATHWAY, TNFA_SIGNALING_VIA_NFKB, INTERFERON_ALPHA_RESPONSE, INFLAMMATORY_RESPONSE, and COMPLEMENT COAGULATION were induced in individuals at high risk. While PI3K_AKT_MTOR_SIGNALING, BILE_ACID_METABOLISM, PROTEIN_SECRETION, SPERMATOGENESIS, FATTY_ACID_METABOLISM, PEROXISOME, PANCREAS_BETA_CELLS, ANDROGEN_RESPONSE and WNT_BETA_CATENIN_SIGNALING were triggered by the group of low-risk patients (**Figure 6A**). Furthermore, the signature also modulated a range of immunologic features associated with the immune system, such as IL2_STAT5_SIGNALING, IL6_JAK_STAT3_SIGNALING, NOTCH_SIGNALING, etc., indicating possible role of laRlncRNA pair-based signature in immunity (79–81).

### laRlncRNA Pairs-Based Risk Signature and Immune Characteristics

**Figure 7:**
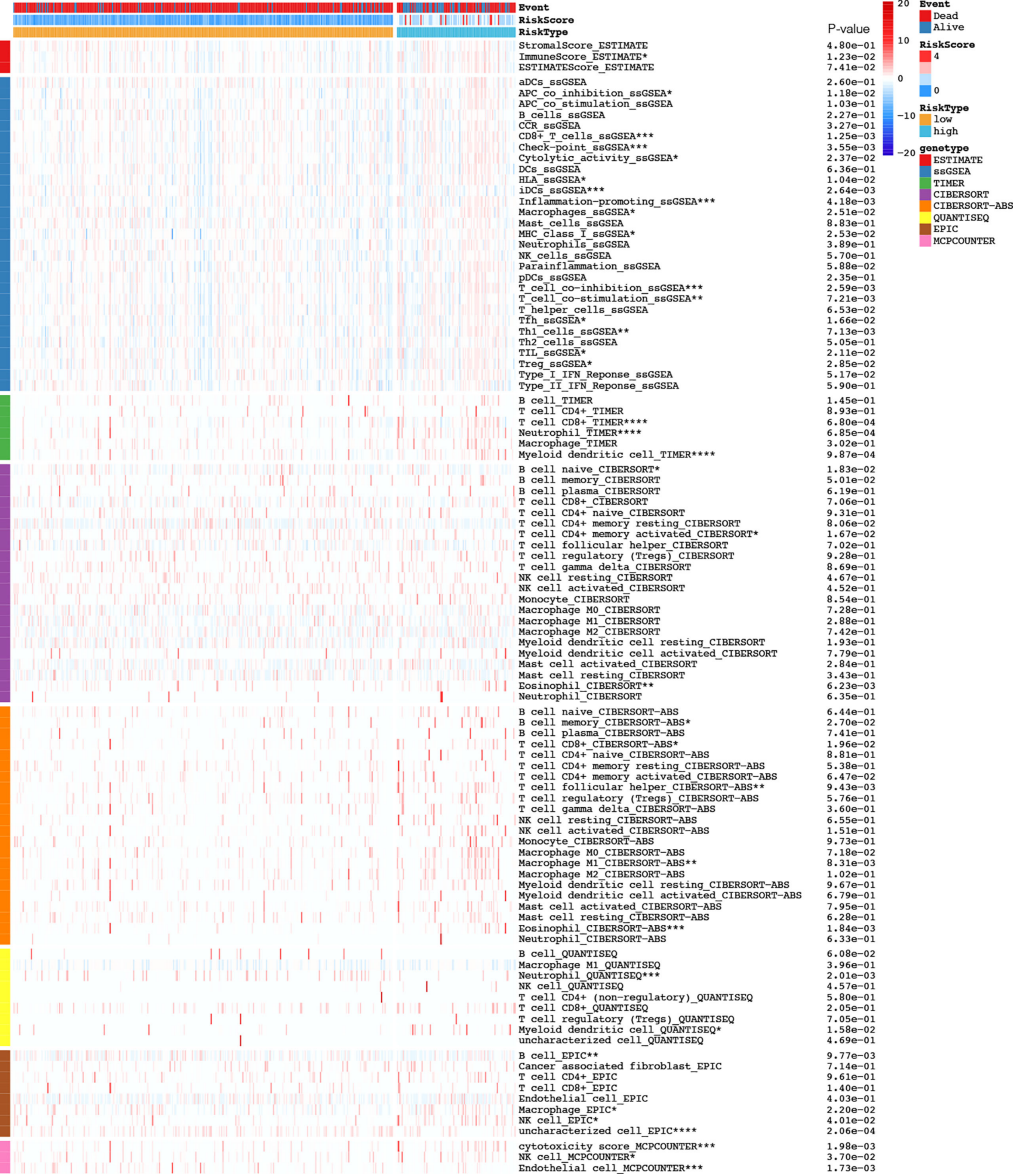
Based on CIBERSORT, CIBERSORT-ABS, EPIC, ESTIMATE, MCP counters, QUANTISEQ, TIMER and ssGSEA algorithms, heatmap of immune infiltration in the high- and low-risk groups was showed.

Following the construction of the laRlncRNA pairs-based risk signature, we examined its connection with immune characteristics in COAD. To begin with, TIMER results were used to investigate whether laRlncRNA pairs-based signature was related to TIICs. **Figure 6B** demonstrated a significant positive correlation between the risk score and many types of immune cells. More specifically, the risk score was significantly correlated with the immune infiltration of neutrophils (COR =0.243, P < 3.919e-07), myeloid dendritic cells (COR = 0.248, P < 2.432e-07), CD8+ T cells (COR = 0.186, P < 1.196e-04), and macrophage cells (COR = 0.117, P = 0.016). **Figure 7** is a visualization of the heatmap of immune cells infiltration created by recognized methods, including CIBERSORT, CIBERSORT-ABS, EPIC, ESTIMATE, MCP counters, QUANTISEQ, TIMER and ssGSEA algorithms. The data provided by these findings suggested that the laRlncRNA pairs-based signature observed with COAD correlated significantly with microenvironment and immune cell infiltration.

As we previously mentioned, cancer immunotherapy using ICGs has emerged as a promising therapeutic option for COAD patients (74). With the training cohorts, we investigated the potential role of the laRlncRNA pairs-based signature in assessing the immunotherapy efficacy of ICGs in COAD patients by analyzing the association between the signature and seven prevalent ICGs targets (PDCD1, PDCD1LG2, LAG3, HAVCR2, CD274, IDO1, and CTLA-4). An analysis of the differences in the expressions of ICGs in different risk groups was carried out by boxplot plots. The laRlncRNA pairs-based signature was significantly correlated with PDCD1, PDCD1LG2, LAG3, HAVCR2, CD274, and IDO1 expressions, whereas no significant difference was found in CTLA4 expression between groups, suggesting that the laRlncRNA pairs-based signature may have a role in predicting the therapeutic response to ICIs immunotherapy in COAD patients. Furthermore, PDCD1, PDCD1LG2, LAG3, HAVCR2, CD274, and IDO1 expression levels were significantly higher in high-risk group than those in the low-risk group (**Figure 6C**).

There is increasing evidence that patients of high microsatellites instability (MSI-H) levels may respond better to immunotherapy and could reap the benefits of immunotheraputics (82). There are yet few studies detecting the significance of the risk score with MSI in COAD. The results of our analysis indicated that there was a significant correlation between a high-risk score and MSI-H status, whereas the microsatellite stable (MSS) status was related to a low-risk score (**Figure 6D**).

TMB is a feature of genomic alterations in tumor cells, which can promote immune recognition and reflect immunotherapy responses (83). Nevertheless, there is a lack of relevant report concerning the risk score and TMB. Accordingly, we examined mutation data obtained from the TCGA-COAD cohort. There was a higher TMB in the high-risk score group compared to that of the low-risk score group (p=0.0081; **Figure 6E** **)**, suggesting that immunotherapy can be beneficial to the patients with high risk scores. Following that, **Figure 6F** depicts the oncoplot of tumor somatic mutations. The top three mutated genes were the same in both risk groups, yet with differences in their mutation frequencies; among them, APC in the low-risk group ranked the first with a proportion of 76%. Specifically, APC (65%), TP53 (55%), and TTN (53%) were the top 3 genes with the highest mutation frequencies in the high-risk group, while in the low-risk group, the top 3 genes were APC (76%), TP53 (54%), and TTN (51%).

### Chemosensitivity Determined in COAD Patients Using Risk Scores

**Figure 8:**
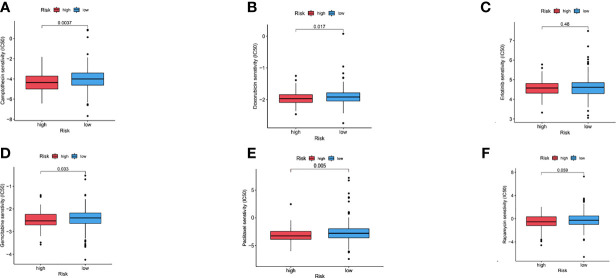
**(A–F)** Chemosensitivity between different risk groups (Camptothecin, Doxorubicin, Erlotinib, Gemcitabine, Paclitaxel, Rapamycin).

It was also tested whether the risk signature can be used to predict chemotherapeutic success for patients with COAD. By comparing IC50 values in high-risk and low-risk groups, Wilcoxon signed-rank test was used to evaluate chemosensitivity. Chemotherapeutic agents exhibited varying sensitivity between different risk groups (p= 0.0037 for Camptothecin, p=0.005 for Paclitaxel, p=0.0017 for Doxorubicin, p=0.48 for Erlotinib, p=0.033 for Gemcitabine, and p=0.059 for Rapamycin). At the same time, it was revealed that an increased IC50 value of several chemotherapeutics (i.e., Camptothecin, Paclitaxel, Doxorubicin, and Gemcitabine) was significantly correlated with a lower risk score (**Figure 8**), thereby suggesting that the laRlncRNA pairs-based risk signature could predict chemotherapy effectiveness in patients with COAD. Hence, results of these study may provide new references for the treatment of COAD in the clinical setting.

### Differences in laRlncRNA Expression in Colon Cancer Cell Lines and Tissues

**Figure 9:**
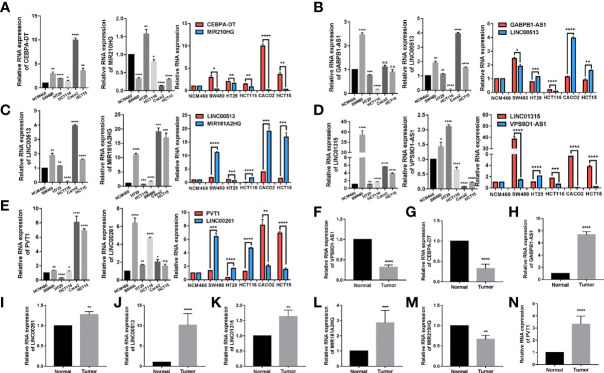
qRT-PCR validation of lncRNA expression levels in different tissues and cell lines. The expression levels of **(A)** CEBPA-DTM|R210HG, **(B)** GABPB1-AS1|LINC00513, **(C)** LINC00513|MIR181A2HG, **(D)**. LINC01315|VPS9D1-AS1 and **(E)** PVT1|LINC00261 in different cell lines (NCM460, SW480, Caco2, HCT15, HCT116, and HT29) were measured. **(F–N)** The expression levels of these lncRNAs in patients of colon cancer and their adjacent normal tissues (N=4) were measured. Results were normalized to reference gene GAPDH. (*P < 0.05, **P < 0.01, ***P < 0.001, ****p < 0.0001; ns, not significant).

qRT–PCR was performed to detect the expressions of relevant laRlncRNAs from 5 different tumor cell lines, and the normal epithelial colon cell line NCM460 for experimental verifications. RNA expression levels have been determined for CEBPA-DT, MIR210HG, LINC00513, MIR181A2HG, GABPB1-AS1, PVT1, LINC00261, LINC01315, and VPS9D1-AS1. qRT–PCR experiments showed that CEBPA-DT, LINC00261 and LINC01315 expressions in colon cancer cells were significantly higher than those in NCM460 cells (**Figures 9A–E**). Moreover, the expression levels of laRlncRNAs and laRlncRNA pairs were displayed and differed significantly. In addition to this, we collected 4 cases of colon cancer along with adjacent normal tissue specimens, revealing that VPS9D1-AS, CEBPA-DT and MIR210HG were significantly higher expressed in adjacent normal tissues than cancer ones while the other 6 laRlncRNAs demonstrated opposite results (**Figures 9F–N**).

### LINC01315 Promotes Colon Cancer Cells Proliferation and Migration *In Vitro*

As a means of investigating the biological role of the lncRNAs, we conducted molecular validations through functional experiments. Amid the many candidates in our signature, we selected LINC01315, an entirely new lncRNA that has virtually never been reported in colon cancer. It was found that LINC01315 was significantly higher expressed in colon cancer tissues and cell lines based on qRT-PCR results in **Figure 9D** and **Figure 9K**. This suggests that in-depth research is warranted.

**Figure 10:**
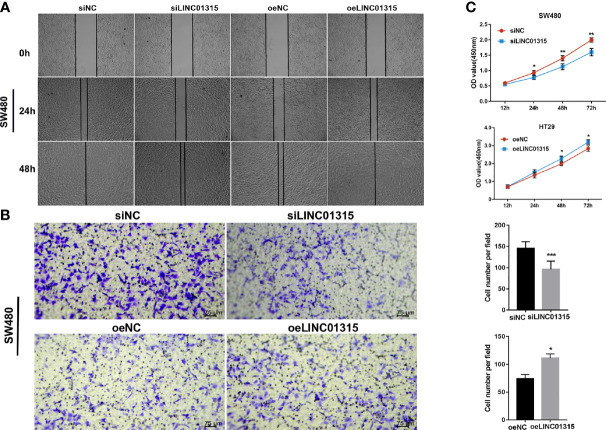
Functional validations of one candidate lncRNA: LINC01315 promotes proliferation and migration of colon cancer cells in vitro. SW480 and HT29 cell were selected and transfected with siLINC01315 and overexpression vector. Evaluation of migration and proliferation capacity by wound healing assay **(A)**, transwell assay **(B)** and CCK-8 assay **(C)**. (*P < 0.05, **P < 0.01, ***P < 0.001).

A total of two groups of cell lines: two knockdown cells (siLINC01315, siNC) and two overexpression cells (oeLINC01315, oeNC) were applied in *in vitro* experiments to study the role of LINC01315 in colon cancer. A selection of SW480 and HT29 cell lines was made. LINC01315 was then tested for its effects on cell migration using wound healing assays (**Figure 10A**) and transwell assays (**Figure 10B**). We first assessed the role of LINC01315 in regulating cell migration by examining wound healing assays. SiLINC01315 cells showed a drastic reduction in migratory ability. OeLINC01315 cells, by contrast, closed the wound area much faster than control cells. Results of transwell chamber assays confirmed this result. Ablation of LINC01315 stimulated proliferation of cells in CCK-8 assays (**Figure 10C**). According to these results, LINC01315 may contribute to colon cancer cells’ proliferation and migration.

## Discussion

COAD has been perceived as an increasing public health and economic threat due to the unclear molecular oncogene diversity at present (84). As has been well established, lactate metabolism plays an influential role in the process of tumor cell growth, metastatic development, and TME (85, 86). Dysregulation of lactate metabolism levels in tumor tissue are detrimentally related to the overall survival in patients with head and neck squamous cell carcinoma (87). However, to the best of our knowledge, there has been little research examining the role of laRlncRNAs and laRlncRNA-based signature in COAD as molecular biomarkers for prognosis and as targets for therapeutic intervention.

Initially, TCGA and GEO databases were used to collect genes expression and clinical information as the training and validation cohorts, from which 37 lactate related DEGs were identified. GO and KEGG analyses suggested that these DEGs were related to the biological functions of organic acid transport, carboxylic acid transport, pyruvate metabolic process, anion transmembrane transporter activity, central carbon metabolism in cancer, mannose type O−glycan biosynthesis, pyruvate metabolism, glycolysis/gluconeogenesis and some cancer-related signaling pathways. These findings are consistent with literature reports that lactate plays a pivotal role in tumors mainly through the acidic environment formed by metabolic process (88, 89).

Next, we conducted a correlation analysis of lactate related DEGs and lncRNAs in the TCGA cohort and identified 2378 laRlncRNAs. According to an unsupervised consensus clustering analysis, COAD patients were categorized into four clusters to ascertain the biological relevance and the potential underlying mechanisms. The immune microenvironment is well recognized as a factor influencing the prognosis and outcomes of immunotherapy in COAD patients (90). The purpose of this study is therefore to correlate the laRlncRNAs with COAD-related tumor immunity. A significant difference in the ESTIMATEScore, particularly in the ImmuneScore, was observed between clusters 1 and other clusters, which indicates that these laRlncRNAs are involved in the tumor immune microenvironment. Meanwhile, we noticed that cluster 1 and the other clusters had a significant difference in tumor purity. Furthermore, in an attempt to elucidate the roles that clusters play in the immune function of COAD patients, further studies were conducted by comparing ICGs and TIICs between clusters. Cluster 1 showed a significantly higher ratio of infiltrations of immune cells. Besides, the expression patterns of ten ICGs displayed significant immune correlation across the four clusters. Cluster 1 differed significantly with cluster 3 and 4 regarding all these ICGs. CD80 and TNFSF1 expression levels were the highest in cluster 2, indicating that this cluster may be more likely to respond to corresponding ICGs-targeted immunotherapy. Thus, our findings indicate that immunity varies between tumor clusters, and further in-depth analysis of laRlncRNAs in COAD is needed to provide insight into the characteristics of immune cell infiltration and anticipations of immunotherapy efficacy.

It has been reported on the role that lncRNA signatures may play in the progression and prediction of survival across various types of cancer, depending upon their specific expression levels (91). In this work, we first developed and tested a new laRlncRNA pairs-based signature in COAD patients by using identified laRlncRNAs whose expression levels were either higher or lower. It reduced the influence of different expression levels on sample errors both in different dataset. LASSO and Cox regression analyses identified five laRlncRNA pairs which are related to prognosis in COAD patients. Additionally, qRT-PCR was conducted to validate the expression of these pairs. LaRlncRNAs, some of which were used for modeling, have already been linked to CRC and other types of diseases. Qiao et al. demonstrated that CEBPA-DT overexpression could inhibit IL-17 signaling to induce the release of cytokines and disruption of immune infiltration, which ultimately enhanced cisplatin resistance to chemotherapy in oral cancer (92). Taheri et al. showed that LINC00513 dysregulation in the peripheral blood of systemic lupus erythematosus patients could be served as a biomarker for diagnosing the disease, tracking the disease’s progression, and assessing therapeutic response (93). Wang et al. suggested that MIR181A2HG, an immune-related lncRNA, was associated with bladder malignancy prognosis and immunotherapy response (94), as well as regulating glucose metabolism and proliferation of human umbilical vein endothelial cells (HUVEC) by regulating AKT2 expression (95). At the same time, we conducted functional experiments of LINC01315, which showed higher expression in colon cancer cell lines and cancer tissues than that in normal controls. Moreover, LINC01315’s biological activity in colon cancer cells was also investigated. We found that it not only promoted proliferation but also enhanced migration of colon cancer cells. Our following research will explore this interesting phenomenon in greater depth. It remains undetermined, however, what mechanisms drive these aforementioned laRlncRNA pairs in COAD prognosis and immunity.

The COAD patients were then classified into high- and low-risk groups according to the calculated cut-off point. As shown by the KM curve, there was a marked difference in the survival rates of low-risk and high-risk patients in the training and validation cohorts. We also conducted univariate and multivariate Cox analyses to evaluate the effectiveness of the risk score and clinical parameters as indicators of patient prognosis. It was concluded that the risk signature served as an independent prognostic predictor for COAD patients. Additionally, subgroup analysis was performed for further validation of the predictive value of the signature for COAD patients based on different clinical characteristics. Stratification analysis of the laRlncRNA pairs-based signature still showed high predictive ability for survival prediction across multiple strata, as determined by patient age, gender, and TNM stage, thus possibly indicating a link with COAD progression and migration. Using our risk model, the calculated AUC value of the risk signature was significantly above that calculated for other clinical characteristics. A nomogram model was further established to determine whether clinical characteristics and risk score influence survival probabilities of patients at 1-, 3-, and 5-year intervals. A calibration graph showed a good matching of the nomogram model predicted survival rates with the actual survival rates, indicating high prediction accuracy. As a result of these findings, the laRlncRNA pairs-based risk signature in COAD may be effective in determining prognosis and defining disease severity, thereby facilitating the implementation and evaluation of our risk model in future clinical practice.

Based on the results of GSVA analysis, it is possible to unearth potential signaling pathways that are implicated in carcinogenesis, including: RESPONSE_TO_OXYGEN_SPECIES_PATHWAY, TNFA_SIGNALING_VIA_NFKB, INFLAMMATORY_RESPONSE at high-risk individuals and PI3K_AKT_MTOR_SIGNALING, WNT_BETA_CATENIN_SIGNALING at low-risk individuals, all of which are common carcinogenic forms that are consistent with published articles (96, 97).

The effectiveness of immunotherapy for lactate metabolism of cancer has been remarkably impressive in recent decades (98, 99). Emerging therapeutic strategies, including PD-1/PD-L1 inhibitors, are used for treating several types of cancers, including colon and lung cancer (100). Therefore, a novel laRlncRNA pairs-based risk signature was developed to investigate the relationship between ICGs and two risk groups as a predictor of immunotherapy response. In our study, this signature correlated with a broad range of ICGs (PDCD1, PDCD1LG2, LAG3, HAVCR2, CD274, IDO1, and CTLA-4), which showed that it might be potentially useful to assess responses to ICGs-targeted therapy. Meanwhile, ICI expression levels in the high-risk group were higher compared to those in the low-risk group, which implied that the laRlncRNA pairs-based signature would be able to predict ICGs expression levels and provide guidance during immunotherapy with ICGs. Frustratingly, this study failed to identify a significant connection between the risk score and CTLA4. In addition, patients with high-risk scores were also more likely to be in MSI-H status and had a higher TMB score. According to our analysis, patients with high-risk score; higher expression of ICGs expression; higher TMB score and MSI-H might respond well to immunotherapy. These findings provide a basis for more comprehensive understanding of anti-tumor immune responses in COAD patients, as well as guidance for personalized immunotherapy treatments.

Moreover, TME, a complex matrix made up of different types of immune cells as well as components related to immunity, has been shown to regulate tumor progression and immunotherapy (101). Previous research suggests that lactate metabolism in the TME influences immune cells infiltration, tumor metastasis, and tumor resistance to different therapies (16). In line with our expectations, a significant association was identified between the laRlncRNA pairs-based risk signature and TME in COAD patients. To examine whether the laRlncRNA pairs-based risk signature was associated with infiltrating immune cells, the following seven approaches were employed: XCELL, TIMER, QUANTISEQ, MCPcounter, EPIC, CIBERSORT-ABS, and CIBERSORT. Aside from this, CD8+ T cells, neutrophils, myeloid dendritic cells, and macrophages were positively related to the risk score, indicating that the signature may contribute significantly to modulating immune cells infiltration (98). Interestingly, the results of this study were also in accordance with earlier report that demonstrated the role of lncRNAs in tumor immunity (102).

Chemotherapy has progressed over the years. So far, one of the major challenges in treating COAD patients is the development of *de novo* or acquired chemoresistance, which may result in curtailed efficacy and adverse prognosis. We thus assessed the chemosensitivity of COAD patients using the laRlncRNA pairs-based risk signature. It was found that a lower risk score was associated with better sensitivity to several chemotherapy drugs, including Camptothecin, Paclitaxel, Doxorubicin, and Gemcitabine. For example, camptothecins (103), alkaloids derived from Camptotheca acuminata that could inhibit DNA topoisomerase I (104), are antitumor agents in the treatment of gastrointestinal cancer, which have been proven to prolong the survival period of early-stage CRC patient by stimulating STAT3 signaling and suppressing PKIP phosphorylation (105). However, resistance to camptothecin clinically remains a mystery (106). The risk signature in our study may function as a promising predictor of chemotherapeutic efficacy, thus enabling the selection of the most appropriate clinical chemotherapy for each patient with COAD.

As far as we know, this is the first laRlncRNA pairs-based risk signature reported for COAD patients. There are, unfortunately, several limitations and drawbacks. It was a small study that relied primarily on TCGA and GEO database data, with the lack of a larger sample size. And the GEO cohort validation was not satisfactory. It was deemed necessary for the prognostic model to be validated on other enormous datasets. For a better understanding of the mechanisms responsible for COAD, additional experiments are necessary to further explore the role of laRlncRNA and immune characteristics. Meanwhile, our study emphasized on bioinformatic analysis based on online datasets, and further validation was performed based on qRT-PCR and several functional experiments, highlighting the necessity of multidimensional molecular mechanism verification in the future. Lastly, further research is needed to recruit more immunotherapy cohorts to strengthen the stability and accuracy of the established signature, so as to determine whether it can be applied to predict resistance to therapeutic agents in future clinical practice.

## Conclusion

The present study systematically describes a novel laRlncRNA pairs-based risk signature constructed for COAD and discusses the potential functions and clinical indications. It is significantly correlated with immune characteristics and can potentially be used to assess individualized risk stratification, personalized antitumor therapeutic efficacy and patients’ survival outcomes. A deeper understanding of the underlying mechanisms of this laRlncRNA pairs-based risk signature is necessary to facilitate individualized treatment of patients with COAD.

## Data Availability Statement

The original contributions presented in the study are included in the article/**Supplementary Material**. Further inquiries can be directed to the corresponding authors.

## Ethics Statement

Informed consent was obtained. This study was approved by the Xiangya Hospital, Central South University Ethics Committee.

## Author Contributions

JX designed the flowchart, analyze the data, and wrote the article. XW oversaw experimental verifications and most of the revision process. YL and XL assisted in data analysis, construction of tables and figures, and critical revision of the gramma. JY and JH supervised the whole process and helped in the critical revision of this manuscript. All authors contributed to the article and approved the submitted version.

## Funding

This study was supported by the Natural Science Foundation of Hunan Province (No. 2020JJ5941 to JY) and Science and Technology Bureau, Changsha (No. kq2001052 to JY).

## Conflict of Interest

The authors declare that the research was conducted in the absence of any commercial or financial relationships that could be construed as a potential conflict of interest.

## Publisher’s Note

All claims expressed in this article are solely those of the authors and do not necessarily represent those of their affiliated organizations, or those of the publisher, the editors and the reviewers. Any product that may be evaluated in this article, or claim that may be made by its manufacturer, is not guaranteed or endorsed by the publisher.
