# Supplementary material for: Lactate Metabolism-Associated lncRNA Pairs: A Prognostic Signature to Reveal the Immunological Landscape and Mediate Therapeutic Response in Patients With Colon Adenocarcinoma
Source: Front Immunol. 2022 Jul 11;13:881359. doi: 10.3389/fimmu.2022.881359 (PMC9328180; doi:10.3389/fimmu.2022.881359)
Supplement: Supplementary file 3 [file DataSheet_3.doc]

**Table S3.** Primers and siRNA sequences used in this study

| Name | Sequence 5'-3' |
| --- | --- |
| LINC01315-forward | CAATTCCCCAGCGTTTTCCC |
| LINC01315-reverse | GGCATCCACTTCATCGCTCA |
| LINC01315-siRNA-forward | CCGGAAAUCUGAUGGGCUUTT |
| LINC01315-siRNA-reverse | AAGCCCAUCAGAUUUCCGGTT |
| LINC00513-forward | CAGTCTGCAGATCCGTCTGG |
| LINC00513-reverse | GCTTAGCGAGGTTGGGTGAT |
| MIR181A2HG-forward | CGCGGTTCAATACCTCGTCT |
| MIR181A2HG-reverse | ATGGGAGAAAGCAGCAACGA |
| CEBPA-DT-forward | GCCCTCAAGTGTCTCCTGTC |
| CEBPA-DT-reverse | CACACATTCATCAGCCCCCT |
| MIR210HG-forward | AGTTCCTGTTGCCAAGCTGA |
| MIR210HG-reverse | GGATGGTCCTGTTGGCTGAA |
| VPS9D1-AS1-forward | CATGGGTAACCAGGGGTCAA |
| VPS9D1-AS1-reverse | GTAAAACGGCCACTTCCCAGA |
| LINC00261-forward | GACATTTGGTAGCCCGTGGA |
| LINC00261-reverse | CCTGCACTCCATGAACACCT |
| GABPB1-AS1-forward | TTCTCGGCGGCGAAGTCTTT |
| GABPB1-AS1-reverse | AGCTTGACTCACTCGCACAC |
| PVT1-forward | CTGTGACCTGTGGAGACACG |
| PVT1-reverse | GCCATCTTGAGGGGCATCTT |
