# Supplementary figures and images for: Lactate Metabolism-Associated lncRNA Pairs: A Prognostic Signature to Reveal the Immunological Landscape and Mediate Therapeutic Response in Patients With Colon Adenocarcinoma
Source: Front Immunol. 2022 Jul 11;13:881359. doi: 10.3389/fimmu.2022.881359 (PMC9328180; doi:10.3389/fimmu.2022.881359)

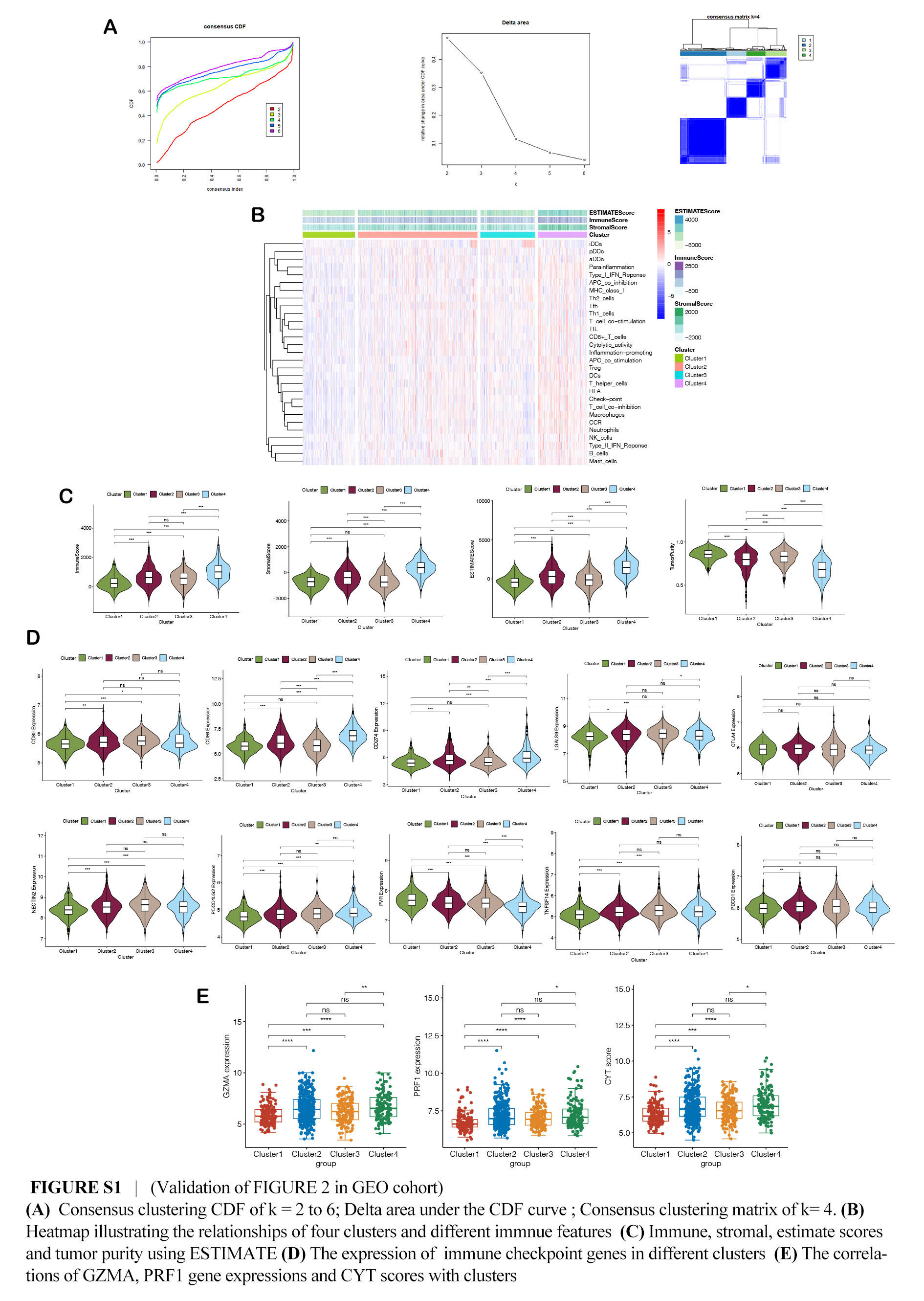

Supplement: Supplementary file 10 [file Image_1.tif]

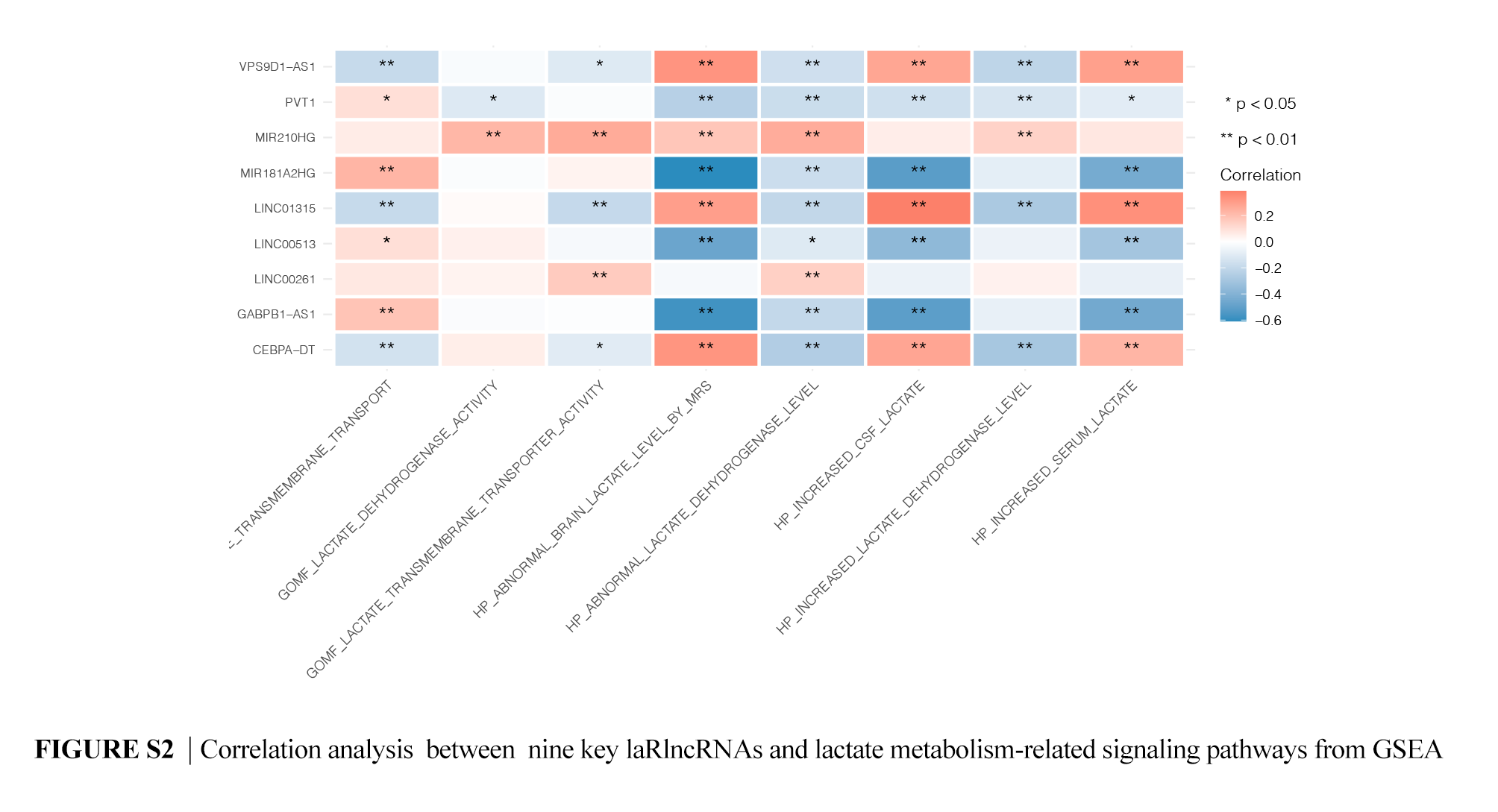

Supplement: Supplementary file 11 [file Image_2.tif]

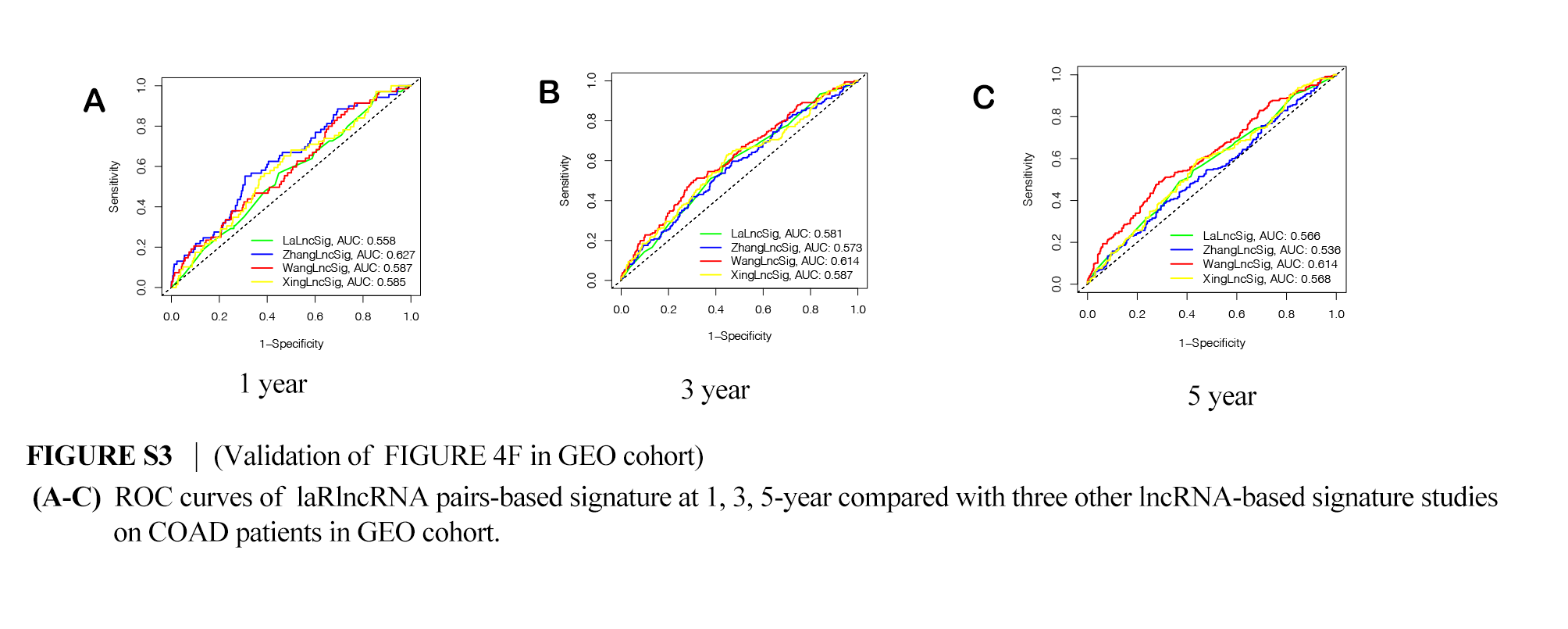

Supplement: Supplementary file 12 [file Image_3.tif]

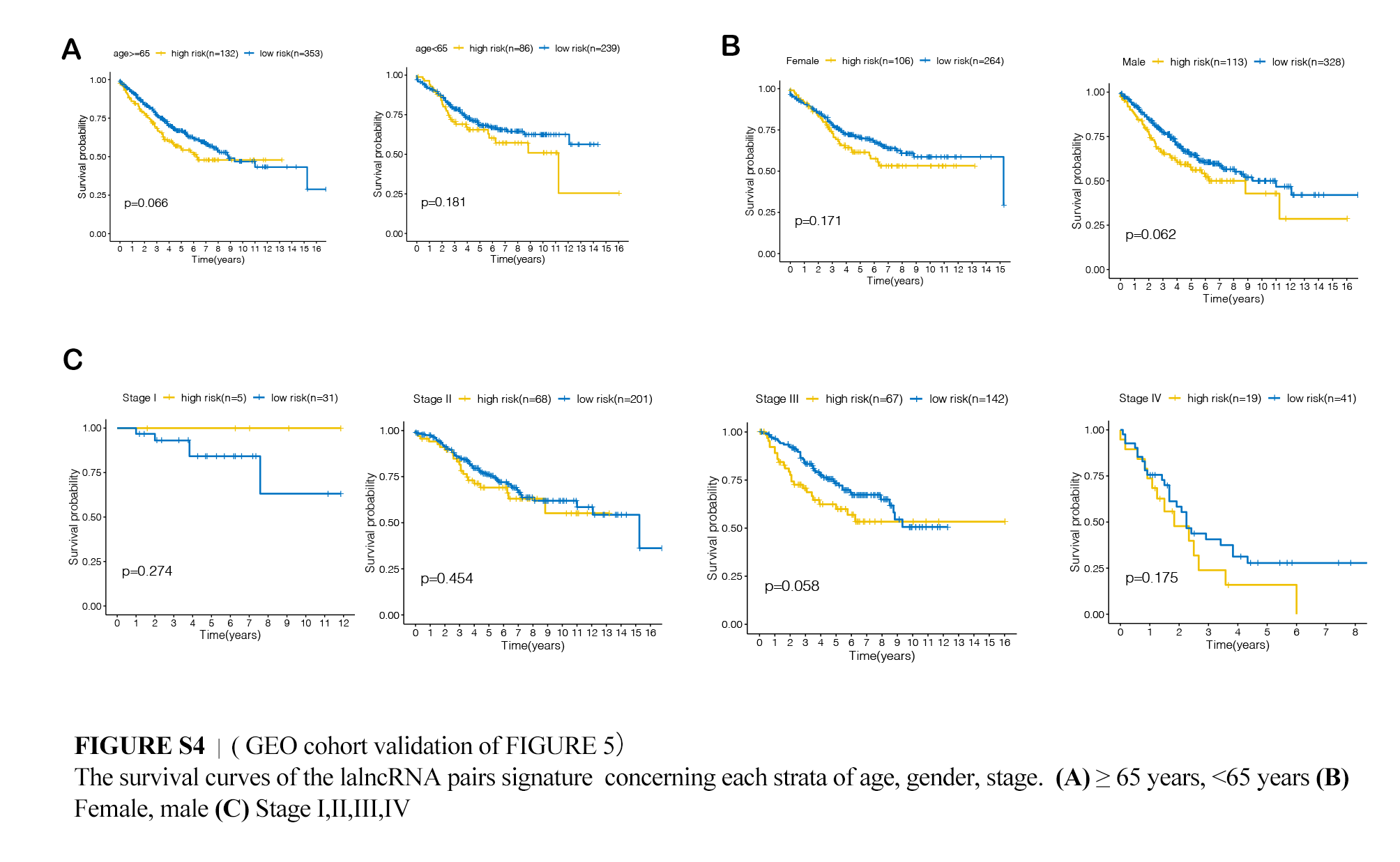

Supplement: Supplementary file 13 [file Image_4.tif]
